# Revealing patterns of endemism in the transatlantic family Chelodesmidae (Polydesmida: Diplopoda)

**DOI:** 10.1111/cla.70022

**Published:** 2025-12-30

**Authors:** Rodrigo Salvador Bouzan, Jackson Means, Kaloyan Ivanov, Antonio Domingos Brescovit, Luiz Felipe Moretti Iniesta

**Affiliations:** ^1^ Laboratório de Coleções Zoológicas Instituto Butantan São Paulo SP Brazil; ^2^ Pós‐Graduação em Zoologia, Instituto de Biociências, Universidade de São Paulo São Paulo SP Brazil; ^3^ Virginia Museum of Natural History Martinsville VA USA; ^4^ Laboratório de Estudos em Diplopoda, Departamento de Zoologia Universidade Federal de Pernambuco Recife PE Brazil

## Abstract

With fossil records dating back to the Silurian/Late Ordovician, millipedes stand out as one of the earliest terrestrial animal groups. Their limited vagility and high endemism make them valuable tools for formulating and testing biogeographic hypotheses, including those related to macro‐vicariance events. Among the order Polydesmida, the family Chelodesmidae displays an intriguing transatlantic distribution, suggesting a Gondwanan origin. Despite this, the evolutionary relationships and biogeography of the family remain largely unknown. In this study, we explore areas of endemism (AEs) for Chelodesmidae using endemicity analysis (NDM/VNDM), utilising a data set of 1512 records for 725 species. NDM/VNDM analyses identified 135 areas of endemism, which were consolidated into 10 generalised areas. These overlapping patterns revealed common areas of endemism, including Central Africa, Western‐Central Africa, Western Africa, West Indies, Northern Amazon, Northern Andes, Guiana Shield, Southern Amazon, America Platina and the Southeastern Mountain Range. This study represents the first explicit assessment of endemism patterns in the family using a quantitative methodology and underscores its significance for further research on Gondwanan distribution patterns.

## Introduction

With fossil records extending to the Silurian (~400 Myr; Wilson and Anderson, 2004) and even the Late Ordovician (~500 Myr; Wilson, 2006), millipedes (class Diplopoda) stand out as one of the earliest terrestrial animal groups, predating the formation of Pangea. Millipedes are known for their limited vagility and high rates of endemism, with populations often confined to mountains, islands or patches of forest (Golovatch and Kime, 2009; Enghoff, 2015). These features make them valuable predictors for formulating and testing biogeographic hypotheses, including those related to macro‐vicariance events (Simonsen, 1990, 1992; Wesener and VandenSpiegel, 2009; Enghoff, 2015; Means and Marek, 2017; Reip and Wesener, 2018; Means et al., 2021; Iniesta et al., 2023a).

Diplopoda are divided into 16 orders distributed across all continents, except Antarctica (Enghoff et al., 2015). The order Polydesmida comprises over 5000 species and is one of the most easily recognisable groups within the class due to the presence of lateral projections on their body rings known as ‘paraterga/paranota’. Polydesmida are distributed across all non‐Antarctic zoogeographic regions and are predominantly found in temperate, subtropical and tropical forests (Shelley and Golovatch, 2011; Enghoff, 2015). The fossil record of the group extends to the Cenozoic (~65 Myr) and is represented by Chelodesmidae, Platyrhacidae, Polydesmidae and Pyrgodesmidae amber fossils (see Edgecombe, 2015).

**Fig. 1:**
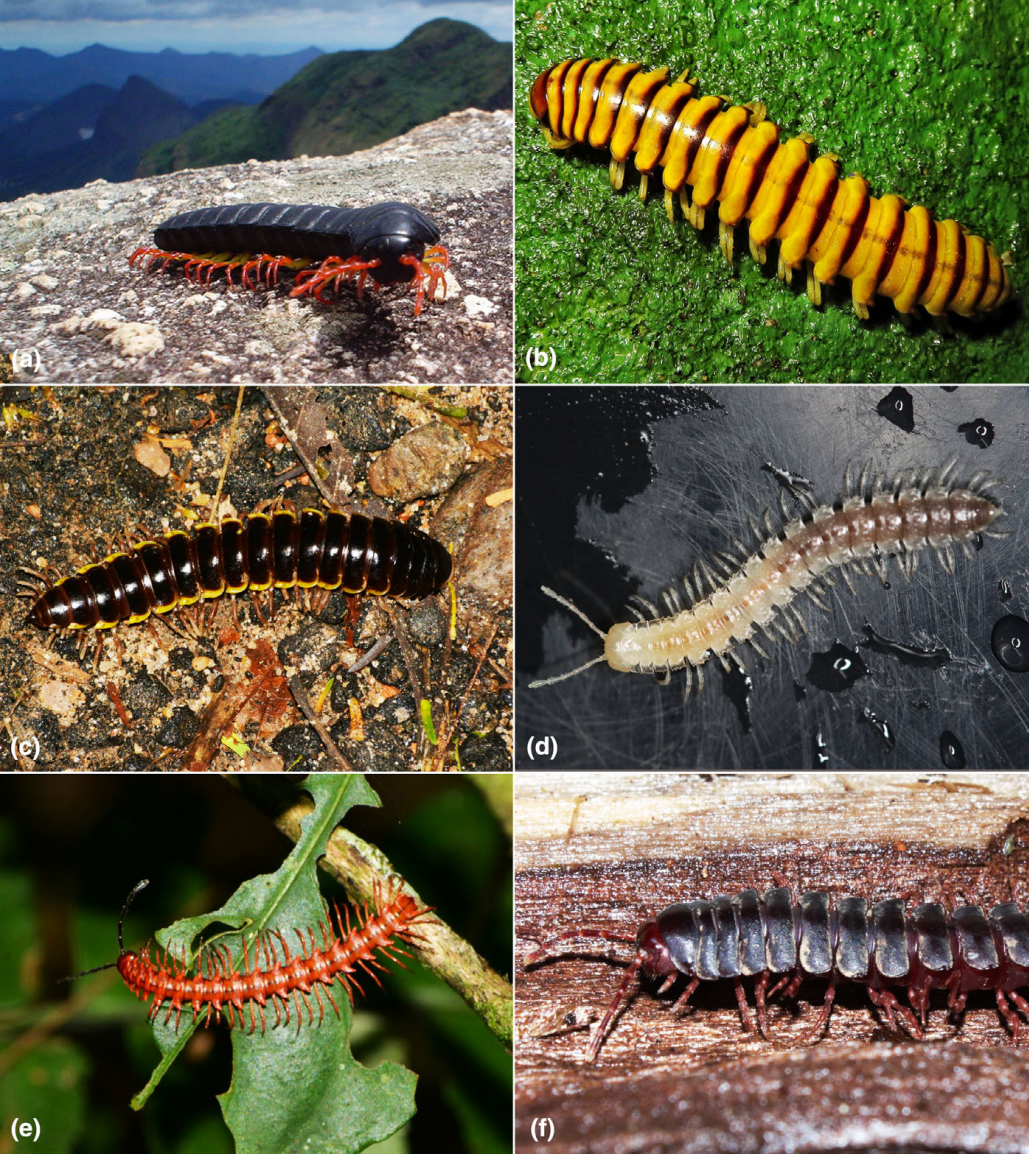
Representatives of the family Chelodesmidae. (a) *Henrisaussurea* sp. (Photo: Edvandro A. Ribeiro); (b) *Eurydesmus* sp. (Photo: Rogerio Dias); (c) *Eurydesmus* sp. (Photo: Edvandro A. Ribeiro); (d) *Cayenniola albaserrata* Bouzan & Iniesta, 2024 (Photo: Rodrigo L. Ferreira); (e) *Obiricodesmus rosascens* (Brandt, 1839) (Photo: Edvandro A. Ribeiro); (f) Chelodesminae sp. (Photo: Weverton dos Santos Azevedo).

With 781 species in 177 genera, Chelodesmidae are one of the most speciose groups within Diplopoda (Hoffman, 1980; Bouzan et al., in press). Members of the family (Fig. 1) exhibit remarkable morphological diversity, including a wide range of colour patterns, paranotal ornamentation and gonopodal structures (Schubart, 1955; Hoffman, 1969; Pena‐Barbosa et al., 2013; Bouzan et al., 2017). Body size varies from medium (~20 mm) to very large (>80 mm), with *Odontopeltis giganteus* (Schubart, 1949) reaching up to 107 mm in length (Pena‐Barbosa et al., 2013). The family primarily inhabits tropical and temperate forests, with some taxa occurring in montane regions of Western Africa and the Andes, dry areas of northeastern Brazil and karst regions of different lithologies (Bouzan et al., 2024a). Most species dwell in the litter layer or beneath logs and stones in humid microhabitats, although vertical migrations and arboreal habits have been observed in Amazonian and Atlantic Forest taxa, respectively. Troglobitic species exhibit typical adaptations to subterranean life, such as depigmentation, elongation of appendages and cuticular thinning, with 27 described cave‐dwelling taxa, seven of which are troglobitic, in 11 countries (Bouzan et al., 2022, 2024a,b; Romero‐Rincon et al., 2025).

Phylogenetically, Chelodesmidae belong to the suborder Chelodesmidea, characterised by the gonopodal coxa positioned externally to the aperture (Simonsen, 1990), which is supported as sister to Polydesmidea + Dalodesmidea. Although numerous taxonomic arrangements of Polydesmida have been proposed (Cook and Collins, 1895; Silvestri, 1897; Attems, 1898, 1899, 1937, 1938, 1940; Brölemann, 1902, 1916; Verhoeff, 1941; Jeekel, 1971; Hoffman, 1980; Shelley, 2003), few have been tested within a phylogenetic framework. The scarcity of systematic and molecular data have hindered the reconstruction of relationships within the group, leaving the classification of many taxa provisional (Means and Marek, 2017). Currently, the family is divided into two subfamilies, Chelodesminae for the Neotropical taxa and Prepodesminae for the Afrotropical and Palaearctic taxa (Hoffman, 1980; Bouzan et al., in press). Chelodesminae comprise 19 tribes, 140 genera with 638 extant and one fossil species, whereas Prepodesminae include two tribes, 37 genera with 142 extant species (Bouzan et al., in press). Recent phylotranscriptomic analyses (Benavides et al., 2023) indicate that Polydesmida originated in the Early Cretaceous (~98 Ma), suggesting a possible Gondwanan origin for Chelodesmidae and subsequent diversification following the fragmentation of the supercontinent. Despite its diversity and intriguing biogeographic pattern, investigations into the evolutionary relationships and biogeography of the family remain largely unexplored. To date, only four phylogenetic studies have been published for some members of the group, none of which have explicitly examined biogeographic or ecological patterns (Pena‐Barbosa et al., 2013; Bouzan et al., 2019, 2021a; Nzoko Fiemapong et al., 2025).

Various methods have been proposed to elucidate distribution patterns and identify areas of endemism (AEs) for different taxa (Morrone, 1994; Szumik et al., 2002; Szumik and Goloboff, 2004; Aagesen et al., 2013). AEs correspond to regions in which taxa share a common distribution due to historical events and ecological factors that constrain the distribution of local species (Nelson and Platnick, 1981; Szumik et al., 2002; Morrone, 2009). AEs can be identified based on the congruent distribution of two or more taxa (Platnick, 1991; Morrone, 1994; Szumik et al., 2002), representing a central concept for the understanding of historical biogeography (Nelson and Platnick, 1981) and for developing hypotheses concerning the biogeographic history of taxa or the regions they inhabit (Cracraft, 1985; Morrone, 1994, 2009; Linder, 2001; Szumik et al., 2002; Domínguez et al., 2006; Ebach et al., 2008; Munguía‐Lino et al., 2016; Della and Prado, 2023).

NDM/VNDM identifies areas of endemism by applying an optimality criterion to evaluate spatial congruence among species distributions, utilising an endemicity index to detect and quantify patterns of endemism. These programmes delineate areas of endemism by utilising grid cells as operational units, without making inferences about relationships or hierarchies between the resulting areas (Szumik et al., 2002; Szumik and Goloboff, 2004).

To date, several endemism studies have been conducted in the Neotropics based on the distribution of insects (e.g., Sigrist and Carvalho, 2008; Ferrari et al., 2010; Garraffoni et al., 2017; Silva and Vaz‐De‐Mello, 2020), spiders (e.g., Sigrist and Carvalho, 2008; Oliveira et al., 2015), harvestmen (e.g., DaSilva et al., 2017), and more recently millipedes (e.g., Iniesta et al., 2023b, c).

We compiled an updated, comprehensive and accessible distributional data set for the millipede family Chelodesmidae to explore its biogeographic patterns and identify AEs using the NDM/VNDM method. This approach is particularly suitable for Chelodesmidae, a group exhibiting high levels of regional endemism, low dispersal ability and strong associations with specific habitats and microclimatic conditions. The use of NDM/VNDM allows the recognition of spatially congruent patterns of endemism, providing a robust framework to evaluate how geological history and ecological constraints have shaped the distribution of Chelodesmidae across the Neotropics and Afrotropics.

## Material and methods

### Dataset

We compiled a data set of global chelodesmid species occurrence records through a comprehensive search of the literature published prior to 2023 (Appendix S1). We did not utilise online databases (e.g., GBIF, iNaturalist) due to difficulties in verifying species‐level identifications using photographs or digitised museum records, and included only records published in the literature, thereby minimising the risk of misidentifications. Data were checked to eliminate repetitive samples, and any samples with imprecise or incongruent locality information were not included in the analyses. Locality information and geographical coordinates were verified in Google Earth Pro (Alphabet, California, USA, v.7.1).

### Areas of endemism

**Fig. 2:**
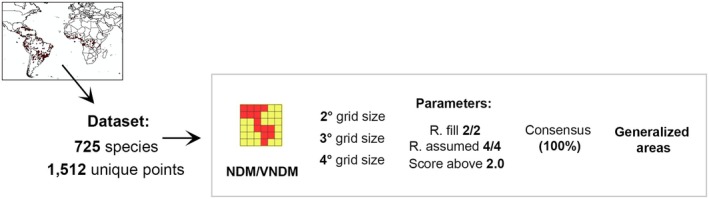
Workflow of the Chelodesmidae endemism analyses.

The dataset was analysed with NDM using grid sizes of 2°, 3° and 4° in VNDM v. 3.0 (Fig. 2; Szumik and Goloboff, 2004). Analyses with different grid sizes were conducted to check the effectiveness of this parameter on patterns of endemism (Aagesen et al., 2009; Escalante et al., 2010, 2013). Searches were conducted using the parameters r. fill = 2/2 and r. assumed = 4/4, which define the extrapolation of species distributions, retaining areas with scores equal to or above 2.0 and containing at least two endemic species. Searches were repeated 100 times with a random seed of zero, keeping overlapping areas only if 90% of the species in each area were unique. Consensus areas (CAs) of endemism were computed using a cut‐off of 100% similarity and strict consensus (Szumik and Goloboff, 2004). Maps were generated by converting the different NDM/VNDM outputs (.txt format) into shape files (.shp format) for each grid size in DIVA‐GIS 7.5.0.

## Results

The final data set comprised a total of 1512 occurrence records for 725 species corresponding to an average of 2.08 records per species. The data set included 474 (65.4%) species with a single occurrence record, 125 (17.2%) species with two records and 126 (17.4%) species with three or more records. The two most common species were the Neotropical *Brasilodesmus paulistus* (Brölemann, 1902) and the Mediterranean Palaearctic *Macellolophus rubromarginatus* (Lucas, 1846), with 34 (2.25%) occurrence records each. The subfamily Chelodesminae accounted for 1216 (80.4%) occurrence records, whereas Prepodesminae accounted for 296 (19.6%) records.

**Fig. 3:**
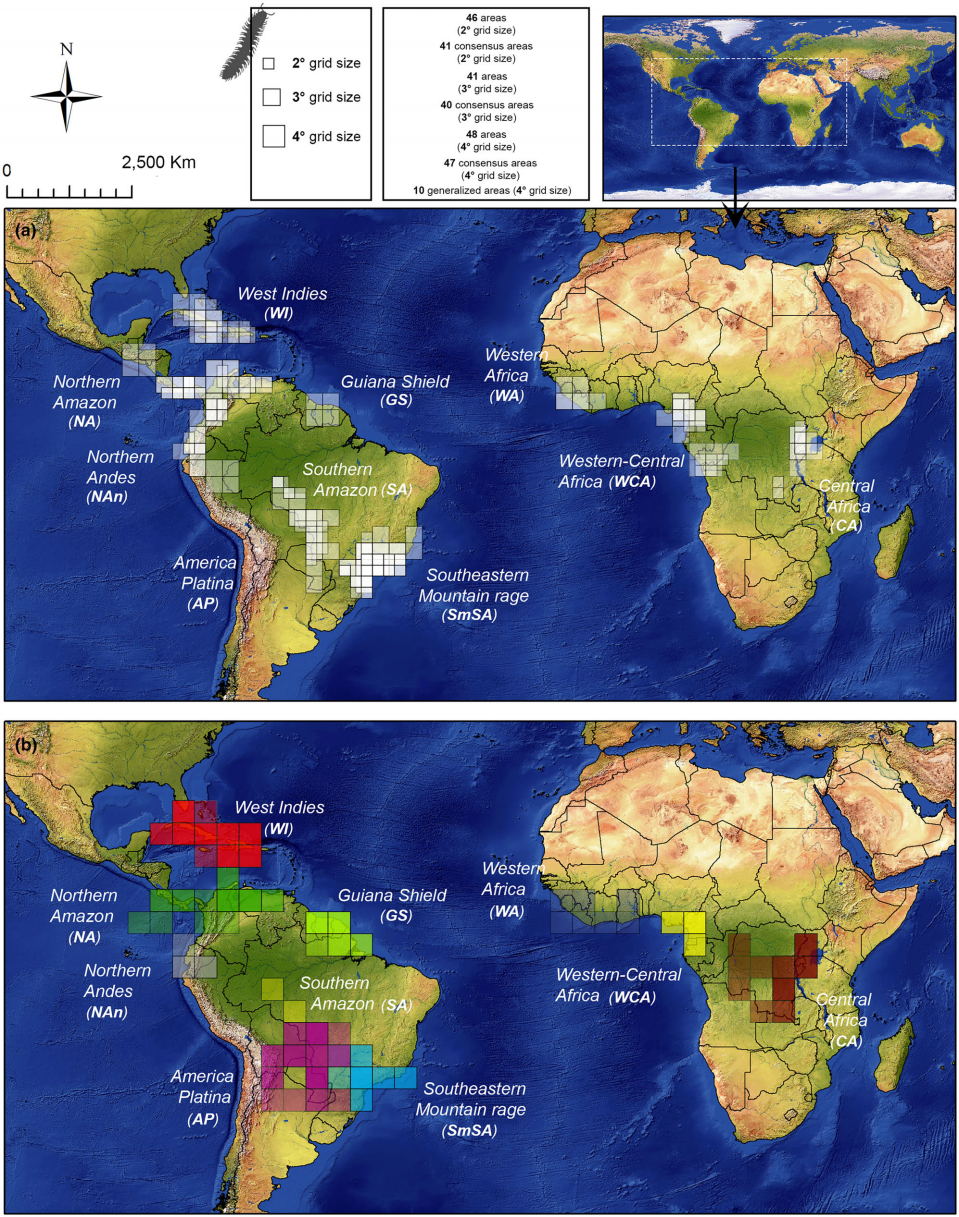
Chelodesmidae areas of endemism obtained through NDM/VNDM. (a) Areas based on 2° and 3° grid size; (b) Areas based on 4° grid. Complete results are available in Appendix S2.

The NDM/VNDM analyses recovered a total of 135 areas and 128 consensus areas including 41 CAs for the 2° grid, 40 CAs for the 3° grid and 47 CAs for the 4° grid (see Appendix S2 for complete results). For ease of visualisation and based on the overlapping patterns between these areas, 10 generalised areas were recognised (Fig. 3): 1. Central Africa (CA), 2. Western‐Central Africa (WCA), 3. Western Africa (WA), 4. West Indies (WI), 5. Northern Amazon (NA), 6. Northern Andes (NAn), 7. Guiana Shield (GS), 8. Southern Amazon (SA), 9. America Platina (AP) and 10. Southeastern Mountain Range (SmSA).

**Fig. 4:**
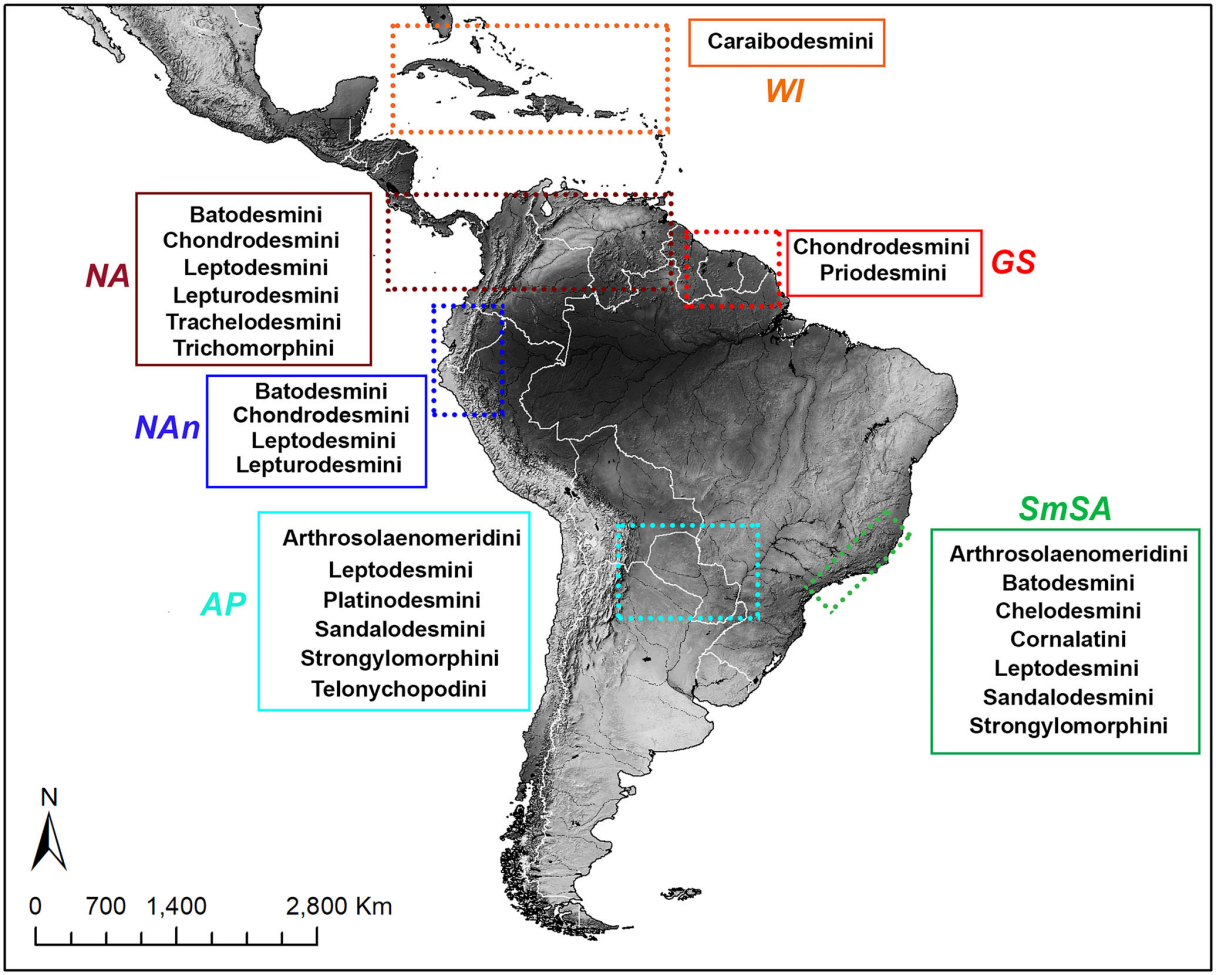
Chelodesmidae areas of endemism related to American tribes obtained through NDM/VNDM.

The assessment of patterns using different grid sizes, overlapped with chelodesmids with assigned tribes, allowed the identification of six areas related to American tribes (Table 1; Fig. 4): 1. West Indies (WI), 2. Northern Amazon (NA), 3. Northern Andes (NAn), 4. Southeastern Mountain range (SmSA), 5. America Platina (AP) and 6. Guiana Shield (GS). No areas were recovered for Prepodesminae tribes.

**Table 1:** Chelodesmidae Areas of Endemism (AEs) by tribe, obtained through NDM/VNDM

Subfamily	Tribe	NDM/VNDM
Chelodesminae	Arthrosolaenomeridini	AP, SmSA
	Batodesmini	NA, NAn, SmSA
	Caraibodesmini	WI
	Chelodesmini	SmSA
	Chondrodesmini	NA, NAn, GS
	Cornalatini	SmSA
	Dibolostethini	–
	Gonorygmatini	–
	Leptodesmini	SmSA, AP, NA, NAn
	Lepturodesmini	NA, NAn
	Macrocoxodesmini	–
	Pandirodesmini	–
	Platinodesmini	AP
	Priodesmini	GS
	Sandalodesmini	SmSA, AP
	Strongylomorphini	SmSA, AP
	Telonychopodini	AP
	Trachelodesmini	NA
	Trichomorphini	NA
Prepodesminae	Thanatomimini	–
	Macellolophini	–

## Discussion

The order Polydesmida, which likely originated in the mid‐Silurian (~420 Myr), comprises several families whose present distribution reflects the fragmentation of Laurasia and Gondwana (Brewer and Bond, 2013). Fossil evidence of Chelodesmidae from Dominican (Miocene; Santiago‐Blay and Poinar, 1992) and Mexican amber (Oligocene–Miocene; Riquelme et al., 2014) confirms the presence of the family since at least the early Cenozoic. Although these fossils do not predate the Cenozoic, recent molecular clock estimates based on a densely sampled phylotranscriptomic data set suggest that Polydesmida diverged during the Cretaceous (~98 Ma), with Chelodesmidae arising shortly thereafter (Benavides et al., 2023). This timeline coincides with the final fragmentation of Gondwana, supporting a biogeographic scenario in which the transatlantic distribution of Chelodesmidae, coupled with its low dispersal ability, reflects an ancient vicariant event. Morphological differentiation between the Neotropical Chelodesminae and the Afrotropical Prepodesminae further reinforces this historical biogeographic separation (Hoffman and Reid, 1990).

The family Chelodesmidae exhibits pronounced microendemism, especially in cave environments, where 27 species are recorded, 7 of which are troglobionts (Bouzan et al., 2022, 2024a; Romero‐Rincon et al., 2025). The concentration of troglobiont Chelodesmidae in the Neotropics, particularly in Brazil, highlights the group's potential as a model for biogeographic inference (Golovatch and Kime, 2009; Enghoff, 2015; Deharveng and Bedos, 2018). Interestingly, none of these troglobitic species was recovered as components of the identified areas of endemism. This likely reflects their extremely localised distributions, which preclude spatial overlap with other taxa and thus prevent their inclusion in broader endemic clusters detected by NDM/VNDM. These findings reinforce the notion that cave‐dwelling species represent highly specialised, microendemic lineages shaped by local isolation, while the recovered areas of endemism capture patterns operating at regional scales.

The generalised distribution pattern of Chelodesmidae includes a South American group from the America Platina (Bolivia, Paraguay) and the southeastern region of Brazil, extending towards the Andes (Ecuador, Peru), the Amazon region–Isthmus of Panama (Brazil, Colombia, Costa Rica, Panama, Venezuela), and the West Indies; and an African group from the Western African region (Guinea, Ivory Coast) extending towards Central Africa (Republic of the Congo, Democratic Republic of the Congo, Uganda, Tanzania) (Figs. 3, 4). These generalised distribution patterns provide the context for interpreting the areas of endemism identified in our analyses.

The endemic areas of the subfamily Chelodesminae are notable for their diversity and prevalence of other endemic millipede groups. One of the most significant areas includes the Southeastern Mountain Range (SmSA), which is renowned for its unique millipede fauna extensively documented during the twentieth century (Brölemann, 1909; Schubart, 1945, 1949, 1950). The region encompasses the Atlantic Forest biome, a global biodiversity hotspot with high levels of endemism threatened by human activities including mining, logging, regional medium and large‐scale farming, and, more recently, extensive urban expansion and industrialisation (Fonseca, 1985; Myers et al., 2000; Colombo and Joly, 2010; Lembi et al., 2020). The area includes mountain ranges (e.g., Serra da Paranapiacaba, Serra da Mantiqueira, Serra do Mar) that emerged during the separation of South America and Africa during the Cretaceous (~100 Myr). These mountain ranges acted as drivers of species diversification, notably observed in the millipede order Spirostreptida (Iniesta et al., 2023a, b, c). Certain chelodesmid tribes, particularly Chelodesmini and Cornalatini, exhibit endemic distributions along these mountainous areas (Table 2; Fig. 4). Members of other tribes (e.g., Arthrosolaenomeridini, Sandalodesmini, Strongylomorphini and Leptodesmini) and genera of uncertain tribal position (e.g., *Atlantodesmus*, *Henrissasurea*, *Heptoporodesmus*, *Hoffmanopeltis*, *Iguazus, Odontopeltis* and *Rupidesmus*) have also been recorded within the areas of endemism recognised in our analysis. Some of these areas were additionally recovered in transitional zones between the Atlantic Forest and Cerrado, reinforcing the biogeographic complexity of southeastern Brazil. However, the extent to which these mountains and ecotonal regions influence peripatric or allopatric speciation of millipedes remains unknown.

**Table 2:** Characteristics of the Chelodesmidae Areas of Endemism (AEs) obtained through NDM/VNDM. (p) indicates *in part*

AEs	Tribe	Country	Characteristics
America Platina (AP)	Arthrosolaenomeridini Leptodesmini Platinodesmini Sandalodesmini Strongylomorphini Telonychopodini	Argentina (p), Bolivia (p), Brazil (p), and Paraguay (p)	The area predominantly encompasses the Chacoan dominion (Chaco and Cerrado provinces) and also includes parts of the South Brazilian (Rondônia province) and Paraná (Esteros del Iberá and Paraná Forest provinces) dominions. It includes the vast plains of the Pampas characterized by grasslands and herbaceous vegetation, while the Brazilian highlands support a mix of forest types, including seasonal and montane forests. The Pantanal consists of seasonal wetlands and flooded grasslands. Major South American rivers such as the Paraná, the Paraguay, and the La Plata, contribute to the region's ecological diversity.
Guiana Shield (GS)	Chondrodesmini Priodesmini	Suriname, French Guiana, Guyana (p) and Brazil (p)	The area covers geologically diverse landforms including lowland plains, coastal areas, and parts of the ancient Guiana Shield. It is covered by dense tropical rainforests and includes major rivers such as the Amazon, the Oyapock and the Maroni.
Northern Amazon (NA)	Batodesmini Chondrodesmini Leptodesmini Lepturodesmini Trachelodesmini Trichomorphini	Venezuela (p), Colombia. Panama and Costa Rica	The area encompasses the Pacific dominion and the Páramo province in the South American Transition Zone. Its diverse geological landscape includes major mountain ranges (e.g., the Andes), coastal plains and river valleys. The vegetation of the area is equally varied and is represented by dense tropical forests, savannas, and mangroves. The Magdalena River in Colombia, the Orinoco River in Venezuela, and the San Juan River in Nicaragua play a crucial role in partitioning endemism and shaping local distribution patterns.
Northern Andes (NAn)	Batodesmini Chondrodesmini Leptodesmini Lepturodesmini	Ecuador and Peru (p)	This geologically diverse area encompasses a transition zone between several dominions (Pacific [Ecuadorian and Western Ecuador provinces], Boreal Brazilian [Napo province], South Brazilian [Ucayali province] and South American Transition Zone [Páramo and Desert provinces]). It includes the Andes and portions of the Amazon basin, including the Marañón, the Pastaza, and the Napo Rivers. The vegetation of the area varies from dense lowland rainforests to cloud forests and paramo grasslands at the higher altitudes.
Southeastern Mountain Range (SmSA)	Arthrosolaenomeridini Batodesmini Chelodesmini Cornalatini Leptodesmini Sandalodesmini Strongylomorphini.	Brazil	The area predominantly covers the Paraná dominion (Araucaria and Parana Forest, and Atlantic provinces) and also includes parts of the Cerrado (Chacoan Dominion). The vegetation is represented by seasonal moist and dry broad‐leaf tropical forests, tropical and subtropical grasslands, savannas, shrublands and mangroves.
West Indies (WI)	Caraibodesmini	The Bahamas, Cuba, Haiti, Dominican Republic, and Jamaica	A geologically complex island system with active volcanoes, atolls and limestone islands, and mountain ranges. The vegetation consists of tropical rainforests in the wet lowlands, tropical and subtropical moist broadleaf and montane forests.
Central Africa (CA)	–	Angola, Democratic Republic of the Congo, Rwanda, Burundi, Uganda and Tanzania	The area predominantly covers the Shaba subregion (Congolian region) and also includes parts of the Zambezian region. Its diverse geological landscape of crystalline shields, sedimentary basins and volcanic formations includes extensive plateaus, large lakes (e.g., Victoria, Kyoga, Albert, Édouard, Kivu, Tanganyika), and mountain ranges such as the Ruwenzori Mountains. The equally diverse vegetation consists of dense tropical forests, savannas and montane forests. Major rivers, such as the Congo and the Kagera River play a crucial role in partitioning endemism and shaping local distribution patterns.
Western Africa (WA)	–	Guinea, Sierra Leone, Liberia, Ivory Coast, Ghana and Togo	The area predominantly covers the Guinea subregion (Congolian region) and a small portion of the Sudanian region and includes coastal plains, hills, mountain ranges, such as the Nimba Mountains and Loma Mountains, as well as major rivers such as the Niger, the Volta and the Cavalla. The vegetation is represented by dense tropical forests, savannas, and coastal mangroves.
Western‐Central Africa (WCA)	–	Nigeria, Cameroon, Equatorial Guinea and Gabon	The area encompasses portions of the Congolian region (parts of the Guinean and Congo subregions). Its diverse geological landscape of crystalline shields, sedimentary basins, and volcanic formations includes coastal plains, plateaus, mountain ranges, such as the Cameroon Mountains, and major rivers such as the Niger, the Benue and the Ogooué. The vegetation is represented by dense tropical forests, savannas and coastal mangroves.

The chelodesmid tribe Platinodesmini is restricted to the America Platina (AP; Table 2; Fig. 4). Hoffman (1981) discussed Platinodesmini's fairly small geographical distribution, suggesting it may be a relic of an ancestral meridional group as evidenced by its distinctive body shape and unique modifications of male sexual characters. A similar distribution pattern is observed in the genus *Pseudonannolene* Silvestri, 1895 (Spirostreptida: Pseudonannolenidae), suggesting a meridional origin from the Platina region (Argentina, Uruguay, southern Brazil), followed by successive dispersal events towards northern South America (Iniesta et al., 2023a). Notably, a high occurrence of members of the tribes Arthrosolaenomeridini and Telonychopodini was recovered within the areas of endemism (AEs) of this region, being consistently detected across all grid sizes, particularly in areas corresponding to the Pantanal. A similar pattern was observed for members of the genus *Leiodesmus*. Additional records for this region included the genera *Euthydesmus*, *Iguazus*, *Leptodesmus* (Leptodesmini), *Odontopeltis*, *Sandalodesmus* (Sandalodesmini) and *Strongylomorpha* (Strongylomorphini), each recovered in at least one grid size. The latter were predominantly associated with AEs located in Bolivia, Paraguay and Argentina.

The Southern Amazon (SA) portion, encompassing Rondônia and northwestern Mato Grosso, represents the southernmost extent of the Amazon Forest and includes ecotonal areas marking the transition to the Cerrado. This region exhibits a fauna typically associated with these transitional environments, represented by members of the genera *Lithobiodesmus*, *Rhicnostethus* and *Rondonaria*. This pattern suggests that these taxa may be adapted to ecotonal environments, reflecting the biotic interchange between the two major biomes. Consistently, the same species were recovered as grouped together in the analyses performed with all three grid sizes.

The Guiana Shield (GS), encompassing species recorded from Suriname, French Guiana and the northern portion of Amapá State, Brazil, was identified only in the analyses using grid sizes 3 and 4, with the smaller grid recovering records exclusively from Suriname. The fauna associated with this area is composed of representatives of the tribes Chondrodesmini and particularly Priodesmini, which appear to be closely linked to the dense tropical rainforests that dominate the region. Such distributional patterns likely reflect the long‐term environmental stability and biogeographic isolation of the Guiana Shield, which has acted as an important centre of endemism in northern South America.

Northern Andes (NAn) encompassing Peru and Ecuador, includes both the Andean range and adjacent portions of the Amazon Basin. AEs recovered is represented by members of the genera *Inconus*, *Biporodesmus* (Batodesmini), *Leptodesmus* (Leptodesmini) and *Guayapeltis*, as well as by some species belonging to the tribes Lepturodesmini and Chondrodesmini. The distribution of these taxa suggests an adaptation to the complex topography and environmental gradients of the Andes–Amazon interface, where habitat heterogeneity likely promotes diversification and endemism.

Northern Amazon (NA) encompassing Colombia, Venezuela, Panama, Costa Rica, Nicaragua, Honduras and Guatemala, exhibits a highly diverse geological landscape that includes major mountain ranges such as the Andes, as well as coastal plains and extensive river valleys. AEs of this generalised region include representatives of the tribes Batodesmini (Colombia, Venezuela), Chondrodesmini (from Colombia northward to Central America), Trichomorphini (Panama, Costa Rica, Venezuela and Colombia), Leptodesmini and Lepturodesmini (Venezuela) and Trachelodesmini (Venezuela, Colombia). Notably, in the analyses using grid size 4, the insular species *Trichomorpha folia* and *T. hyla* were recovered within a distinct area of endemism. This pattern reinforces the role of these regions as dynamic centres of diversification, where complex geological and ecological conditions have fostered multiple independent radiations within Chelodesmidae.

The northernmost endemic area for Chelodesmidae is the West Indies (WI), which contains a single, exclusive tribe, the Caraibodesmini (Table 2; Fig. 4). The tribe includes 12 species of *Caraibodesmus* Chamberlin, 1918, and the monotypic *Platyurodesmus* Loomis, 1977, restricted to Jamaica. The WI has been the focus of many taxonomic studies during the late nineteenth and early twentieth centuries, which have revealed a great diversity of taxa, primarily within the Polydesmida (Pocock, 1894; Loomis, 1933, 1934, 1936, 1937, 1938, 1941a, b; Hoffman, 1999). The WI is recognised for its geographically complex evolutionary history (see Rodriguez‐Silva and Schlupp, 2021), providing a suitable setting for terrestrial colonisation and diversification through vicariance events and, less commonly, through dispersal events from North America, the Greater Antilles and the Northern Andes. As evidenced in our analysis, in addition to the recovery of Caraibodesmini, several other genera exclusive to the Caribbean region were identified, including *Amphelictogon*, *Cubodesmus*, *Achromoporus*, *Hypselodesmus*, *Ebanodesmus*, *Granmadesmus* and *Plicatodesmus*. Representatives of *Amphelictogon*, *Cubodesmus*, *Achromoporus* and *Hypselodesmus* were recovered across all three grid sizes, whereas and *Ebanodesmus, Granmadesmus* and *Plicatodesmus* were recovered only for one grid size.

The Central African (CA) region, encompassing the Democratic Republic of the Congo and Angola, as well as Rwanda, Burundi, Tanzania, Uganda and the Republic of the Congo, comprises a complex landscape characterised by extensive equatorial forests, savannas and highland regions associated with the Albertine Rift. The fauna of the recovered AEs is represented by members of several genera, including *Morphotelus*, *Pimodesmus*, *Lipodesmus*, *Basachanta* and *Paracordyloporus* in the western portion (Democratic Republic of the Congo and Angola), and *Eucordyloporus*, *Mesodesmus*, *Paracordyloporus*, *Pimodesmus*, *Scaptodesmus* and *Morphotelus* predominantly in the eastern portion (Rwanda, Burundi, Tanzania, Uganda and the Democratic Republic of the Congo). This distribution pattern likely reflects the combined influence of the Congo Basin lowlands and the montane systems of the Albertine Rift, which together create a mosaic of habitats that may promote both persistence of ancient lineages and localised diversification.

Western Central African (WCA), encompassing Nigeria, Cameroon, Gabon and Equatorial Guinea, extends southward into Angola, the Republic of Congo and part of the Democratic Republic of Congo. This region includes a heterogeneous landscape dominated by dense tropical rainforests. AEs of this region comprise members of *Cordyloconus*, *Cryptoporatia*, *Diaphorodesmus*, *Neocordyloporus*, *Paracordyloporus* and *Scaptodesmus* in the northern and coastal areas (Nigeria to Gabon), and *Basacantha*, *Mesodesmus* and *Neocordyloporus* in the southern portion (Angola, Congo and the Democratic Republic of the Congo). This pattern highlights the continuity of faunal elements across the Guineo–Congolian forests and the biogeographic role of this region as a core area of endemism and dispersal within sub‐Saharan Africa.

The Western Africa (WA) region, encompassing Guinea, Sierra Leone, Liberia, Togo, Côte d'Ivoire and Ghana, was recovered only in the analyses using grid sizes 3 and 4. This region is characterised by a complex landscape of lowland tropical rainforests, forest–savanna mosaics, and coastal plains. AEs recorded representatives of the genera *Anisodesmus*, *Cheirodesmus*, *Tylodesmus*, *Prepodesmus* and *Afolobina*, each recorded from one or more of these countries. The distribution of these taxa suggests an affinity with the Upper Guinean Forest block that may have acted as a refuge and centre of endemism.

The current understanding of Chelodesmidae diversity is largely due to the taxonomic efforts of R. V. Chamberlin and J. Carl (north‐western South America), H. F. Loomis, C. Suriel and A.R. Pérez‐Asso (Antilles), O. Schubart (primarily eastern Brazil) and R. L. Hoffman (South America and Africa). The focus of these authors' taxonomic efforts on specific regions generates a strong bias and may explain the limited recognition of endemic areas in Africa, or in other Brazilian biomes such as the Caatinga and the Cerrado. According to Oliveira et al. (2016, 2019), most of Brazil's terrestrial biodiversity (including Polydesmida) has a pattern of spatially biased collecting efforts, especially in relation to roadsides. This pattern is especially evident in Chelodesmidae records from the Brazilian states of São Paulo, Minas Gerais and Pará as recently demonstrated by Bouzan et al. (2018, 2021b, 2024b).

With the exception of *Macellolophus* Attems, 1940, and *Cantabrodesmus* Mauriès, 1971, all members of Prepodesminae are exclusively African. Although Western Africa (WA), Western‐Central Africa (WCA) and Central Africa (CA) were recovered as endemic for Prepodesminae (Fig. 3), the overall pattern suggests limited diversification and endemism resolution. This likely reflects the strong sampling bias towards Neotropical taxa, which account for over 80% of all records. Furthermore, African chelodesmids were most extensively studied during the mid‐twentieth century (Hoffman, 1980), however, a large number of taxa remain poorly revised, constraining biogeographic inference, especially when compared to better studied groups, such as the African Gomphodesmidae and Oxydesmidae (Hoffman, 1990, 2005). These patterns highlight that the apparent scarcity of endemism in Africa is likely an artefact of uneven sampling and historical taxonomic effort rather than a true biological signal.

The robustness of the detected areas of endemism should be interpreted with caution, given the high proportion of species represented by a single occurrence (65.4%) and the average of only two records per species. This level of spatial sparsity limits the resolution of NDM/VNDM analyses and may obscure smaller or less congruent areas, as observed in other arthropod data sets (Lim et al., 2012; Hoffmeister and Ferrari, 2016). Nonetheless, the identification of congruent areas across multiple scales supports the reliability of the main patterns.

The species composition within the recovered areas of endemism reveals consistent supraspecific and ecological patterns. In the Neotropics, several areas show a strong concentration of congeners, suggesting in situ diversification. The Southeastern Mountain Range (SmSA) and America Platina (AP) share representatives of the tribes Sandalodesmini, Strongylomorphini and Leptodesmini, as well as the genera *Iguazus* and *Odontopeltis*, indicating faunal continuity and historical connectivity between these adjacent regions. The Guiana Shield (GS) is characterised primarily by members of Chondrodesmini, which is also shared with the Northern Andes (NAn) and Northern Amazon (NA) areas. These latter regions, in turn, include representatives of Batodesmini, Chondrodesmini, Leptodesmini and Lepturodesmini, reflecting a broad biotic interchange across the northern portion of South America. In contrast, the West Indies (WI) harbours a distinct assemblage dominated by the tribe Caraibodesmini, represented by *Caraibodesmus* and *Platyurodesmus*, which are restricted to island habitats and exhibit typical insular endemism. In Africa, the Central African (CA) and Western Central African (WCA) areas share genera such as *Mesodesmus*, *Paracordyloporus*, *Scaptodesmus* and *Basachanta*, suggesting faunal continuity within the Guineo–Congolian region. Altogether, these compositional patterns emphasise the biogeographic structuring of Chelodesmidae and suggest that both historical isolation and ecological specialisation have contributed to the diversification of the family across tropical regions.

This study represents the first explicit assessment of endemism patterns in the family Chelodesmidae using a quantitative methodology for analysing worldwide patterns of endemism, establishing a basis for subsequent biogeographic analyses. Future studies should focus on time‐calibrated phylogenies and detailed morphological analyses to further elucidate the evolutionary history of the group.

## Conflict of interest

The authors declare that they have no conflict of interest.

## Supporting information


**Appendix S1.** Compiled data set of global Chelodesmidae species occurrence records.

**Appendix S2.** Results of the consensus areas identified by the NDM/VNDM analyses.

## Data Availability

The data that support the findings of this study are available from the corresponding author upon reasonable request.
