# Supplementary material for: Revealing patterns of endemism in the transatlantic family Chelodesmidae (Polydesmida: Diplopoda)
Source: Cladistics. 2025 Dec 30;42(2):159–71. doi: 10.1111/cla.70022 (PMC12977946; doi:10.1111/cla.70022)
Supplement: Supplementary file 2 — Appendix S2. Results of the consensus areas identified by the NDM/VNDM analyses. [file CLA-42-159-s001.docx]

| **Grid** | **AoE** | **AoE included** | **AoE**  **cells** | **N° species scored** | **IE (min. - max.)** | **Species/ *E*** |
| --- | --- | --- | --- | --- | --- | --- |
| 2° x 2° | *c* 0 | 0 | 3 | 4 | 3.020833 | *Alocodesmus intermedius* (0.781); *Biporodesmus planus* (0.781); *Plusioporodesmus bellicosus* (0.625); *Chondrodesmus convexus* (0.833) |
|  | *c* 1 | 1 | 3 | 3 | 2.500000 | *Trichomorpha capillata* (0.833); *Trichomorpha crucicola* (0.833); *Trichomorpha esulcata* (0.833) |
|  | *c* 2 | 2 | 3 | 3 | 2.500000 | *Chondrodesmus atrophus* (0.833); *Trichomorpha capillata* (0.833); *Trichomorpha panamica* (0.833) |
|  | *c* 3 | 3 | 3 | 3 | 2.500000 | *Alocodesmus angustatus* (0.833); *Chondrodesmus atrophus* (0.833); *Trichomorpha panamica* (0.833) |
|  | *c* 4 | 4, 6 | 5 | 5 | 3.250000 - 3.777778 | *Eurydesmus alcatrazensis* (0.833-1.000); *Leptodesmus forceps* (0.833-1.000); *Brasilodesmus paulistus paulistus* (0.000-0.333); *Strongylomorpha volutatus* (0.750-1.000); *Henrisaussurea ramosa* (0.500-0.778) |
|  | *c* 5 | 5 | 3 | 5 | 4.148148 | *Eurydesmus herteli* (0.833); *Leptodesmus pubescens* (0.713); *Brasilodesmus paulistus meridionalis* (1.000); *Strongylomorpha araucariae* (1.000); *Atlantodesmus eimeri* (0.602) |
|  | *c* 6 | 7 | 6 | 8 | 5.916667 | *Eurydesmus alcatrazensis* (0.667); *Leptodesmus didymus* (0.667); *Leptodesmus forceps* (0.667); *Leptodesmus rubricus* (0.667); *Brasilodesmus lamellosus* (0.667); *Brasilodesmus paulistus paulistus* (1.000); *Strongylomorpha volutatus* (0.750); *Henrisaussurea ramosa* (0.833) |
|  | *c* 7 | 8 | 3 | 4 | 3.083333 | *Dialysogon jucundus* (0.833); *Leptodesmus vagans* (0.833); *Sandalodesmus gasparae* (0.917); *Henrisaussurea ramosa* (0.500) |
|  | *c* 8 | 9 | 7 | 8 | 5.500000 | *Eurydesmus herteli* (0.643); *Leptodesmus pubescens* (0.750); *Brasilodesmus catharinensis* (0.500); *Brasilodesmus decipiens* (0.750); *Brasilodesmus paulistoides* (0.679); *Brasilodesmus paulistus meridionalis* (0.714); *Strongylomorpha araucariae* (0.714); *Atlantodesmus eimeri* (0.750) |
|  | *c* 9 | 10 | 2 | 10 | 9.583333 | *Alocodesmus mammatus* (1.000); *Eurydesmus angulatus* (1.000); *Cornalatus permutatus* (1.000); *Obiricodesmus terrigena* (1.000); *Leptodesmus carneus* (1.000); *Leptodesmus therezopolis* (1.000); *Strongylomorpha organorum* (1.000); S*trongylomorpha vanvolxemi* (1.000); *Henrisaussurea corcovadis* (0.917); *Odontopeltis tiradentes* (0.667) |
|  | *c* 10 | 11, 14, 16 | 8 | 7 | 5.761905 - 6.428571 | *Amphelictogon alayoni* (0.833-1.000); *Amphelictogon cubanus* (0.648-1.000); *Amphelictogon dentatus* (0.833-1.000); *Amphelictogon obscurus* (0.833-1.000); *Amphelictogon pallidipes* (0.833-1.000); *Cubodesmus prominens* (0.595-1.000); *Cubodesmus ramsdeni* (0.833-1.000) |
|  | *c* 11 | 12, 39 | 8 | 6 | 4.242188 - 4.650000 | *Eurydesmus herteli* (0.750); *Leptodesmus pubescens* (0.609-0.938); *Brasilodesmus decipiens* (0.650-0.938); *Brasilodesmus paulistus meridionalis* (0.563-0.875); *Strongylomorpha araucariae* (0.563-0.875); *Atlantodesmus eimeri* (0.563-0.820) |
|  | *c* 12 | 13 | 2 | 2 | 2.000000 | *Morphotelus rotundalatus* (1.000); *Pimodesmus numerosus* (1.000) |
|  | *c* 13 | 15 | 2 | 2 | 2.000000 | *Caraibodesmus criniger* (1.000); *Caraibodesmus mammatus* (1.000) |
|  | *c* 14 | 17 | 5 | 5 | 3.600000 | *Eucordyloporus cornuatus* (0.700); *Mesodesmus roccatii* (0.700); *Mesodesmus rugifer* (0.800); *Paracordyloporus (Paracordyloporus) moeranus* (0.700); *Scaptodesmus dentatus* (0.700) |
|  | *c* 15 | 18 | 6 | 3 | 2.216667 | *Euthydesmus acicarina* (0.667); *Leiodesmus orlandi* (0.750); *Leiodesmus validus* (0.800) |
|  | *c* 16 | 19, 30 | 13 | 7 | 4.700000 - 4.785714 | *Cordyloconus vitiosus* (0.643-0.667); *Cryptoporatia verrucosa* (0.786-0.833); *Diaphorodesmus dorsicornis* (0.200-0.500); *Neocordyloporus longipes* (0.643-0.667); *Paracordyloporus (Paracordyloporus) porati* (0.929-1.000); *Scaptodesmus granulosa* (0.643-0.667); *Scaptodesmus porati* (0.643-0.667) |
|  | *c* 17 | 20 | 5 | 3 | 2.350000 | *Basacantha tuberculifer* (0.700); *Mesodesmus robustus* (0.700); *Neocordyloporus falcatus* (0.950) |
|  | *c* 18 | 21 | 4 | 5 | 4.000000 | *Alocodesmus intermedius* (0.813); *Biporodesmus planus* (0.813); *Plusioporodesmus bellicosus* (0.875); *Chondrodesmus convexus* (0.750); *Trichomorpha virgata* (0.750) |
|  | *c* 19 | 22 | 3 | 3 | 2.500000 | *Lithobiodesmus xenoporus* (0.833); *Rhicnostethus rondoni* (0.833); *Rondonaria schubarti* (0.833) |
|  | *c* 20 | 23 | 4 | 9 | 6.930556 | *Eurydesmus alcatrazensis* (0.750); *Leptodesmus didymus* (0.750); *Leptodesmus forceps* (0.750); *Leptodesmus rubricus* (0.750); *Leptodesmus vagans* (0.750); *Brasilodesmus lamellosus* (0.750); *Brasilodesmus paulistus paulistus* (0.556); *Strongylomorpha volutatus* (0.875); *Henrisaussurea ramosa* (1.000) |
|  | *c* 21 | 24 | 4 | 4 | 3.000000 | *Alocodesmus angustatus* (0.750); *Chondrodesmus atrophus* (0.750); *Trichomorpha capillata* (0.750); *Trichomorpha panamica* (0.750) |
|  | *c* 22 | 25 | 3 | 3 | 2.500000 | *Alyssa kalobata* (0.833); *Chondrodesmus armatus* (0.833); *Camptomorpha dorsalis* (0.833) |
|  | *c* 23 | 26 | 4 | 15 | 11.160714 | *Alocodesmus mammatus* (0.750); *Eurydesmus angulatus* (0.750); *Eurydesmus angustus* (0.750); *Eurydesmus zebratus* (0.750); *Cornalatus permutatus* (0.750); *Obiricodesmus terrigena* (0.750); *Leptodesmus carneus* (0.750); *Leptodesmus marginatus* (0.813); *Leptodesmus ruidus* (0.750); *Leptodesmus therezopolis* (0.750); *Leptodesmus wygodzinskyi* (0.750); *Strongylomorpha organorum* (0.750); *Strongylomorpha vanvolxemi* (0.750); *Henrisaussurea corcovadis* (0.813); *Odontopeltis tiradentes* (0.536) |
|  | *c* 24 | 27 | 5 | 3 | 2.000000 | *Amphelictogon subterraneus bahamiensis* (0.800); *Amphelictogon subterraneus dolius* (0.700); *Cubodesmus prominens* (0.500) |
|  | *c* 25 | 28 | 2 | 2 | 2.000000 | *Chondrodesmus atrophus* (1.000); *Trichomorpha panamica* (1.000) |
|  | *c* 26 | 29 | 2 | 4 | 3.428571 | *Leptodesmus didymus* (1.000); *Leptodesmus rubricus* (1.000); *Brasilodesmus lamellosus* (1.000); *Henrisaussurea ramosa* (0.429) |
|  | *c* 27 | 31 | 3 | 3 | 2.833333 | *Alocodesmus intermedius* (0.917); *Biporodesmus planus* (0.917); *Plusioporodesmus bellicosus* (1.000) |
|  | *c* 28 | 32 | 2 | 2 | 2.000000 | *Chondrodesmus armatus* (1.000); *Camptomorpha dorsalis* (1.000) |
|  | *c 29* | 33 | 4 | 5 | 3.450000 | *Cordyloconus vitiosus* (0.750); *Cryptoporatia verrucosa* (1.000); *Neocordyloporus longipes* (0.750); *Paracordyloporus (Paracordyloporus) porati* (0.200); *Scaptodesmus porati* (0.750) |
|  | *c 30* | 34 | 2 | 5 | 5.000000 | *Leptodesmus flagellatus* (1.000); *Leptodesmus propinquus* (1.000); *Trichomorpha hoffmani* (1.000); *Trichomorpha lamottei* (1.000); *Trichomorpha spinosa* (1.000) |
|  | *c 31* | 35 | 2 | 2 | 2.000000 | *Trichomorpha crucicola* (1.000); *Trichomorpha esulcata* (1.000) |
|  | *c 32* | 36 | 2 | 2 | 2.000000 | *Lithobiodesmus xenoporus* (1.000); *Rondonaria schubarti* (1.000) |
|  | *c 33* | 37 | 4 | 9 | 7.312500 | *Achromoporus atrechoensis* (0.750); *Achromoporus ebanoverde* (0.875); *Achromoporus elegans* (0.750); *Achromoporus heteromus* (0.750); *Achromoporus magnus* (0.938); *Achromoporus platyurus* (0.938); *Achromoporus surieli* (0.813); *Hypselodesmus assoi* (0.750); *Hypselodesmus subtilissimus* (0.750) |
|  | *c 34* | 38 | 3 | 13 | 10.583333 | *Alocodesmus mammatus* (0.833); *Eurydesmus angulatus* (0.833); *Eurydesmus angustus* (0.833); *Eurydesmus zebratus* (0.833); *Cornalatus permutatus* (0.833); *Obiricodesmus terrigena* (0.833); *Leptodesmus carneus* (0.833); *Leptodesmus therezopolis* (0.833); *Leptodesmus wygodzinskyi* (0.833); *Strongylomorpha organorum* (0.833); *Strongylomorpha vanvolxemi* (0.833); *Henrisaussurea corcovadis* (0.917); *Odontopeltis tiradentes* (0.500) |
|  | *c 35* | 40 | 2 | 2 | 2.000000 | *Eucordyloporus cornuatus* (1.000); *Scaptodesmus dentatus* (1.000) |
|  | *c 36* | 41 | 4 | 3 | 2.500000 | *Arthrosolaenomeris caipora* (0.750); *Arthrosolaenomeris iara* (1.000); *Vanzolegulus limbatus* (0.750) |
|  | *c 37* | 42 | 2 | 4 | 4.000000 | *Chondrodesmus nobilis* (1.000); *Chondrodesmus tamocalanus* (1.000); *Trichomorpha eusema* (1.000); *Trichomorpha eutyla* (1.000) |
|  | *c 38* | 43 | 3 | 3 | 2.537037 | *Dialysogon jucundus* (0.833); *Leptodesmus tridentatus* (0.917); *Sandalodesmus gasparae* (0.787) |
|  | *c 39* | 44 | 2 | 4 | 2.916667 | *Eurydesmus herteli* (1.000); *Brasilodesmus paulistus meridionalis* (0.667); *Strongylomorpha araucariae* (0.667); *Atlantodesmus eimeri* (0.583) |
|  | *c 40* | 45 | 2 | 2 | 2.000000 | *Chondrodesmus voglii* (1.000); *Lepturodesmus meinerti* (1.000) |
| 3° x 3° | *c 0* | 0 | 2 | 2 | 2.000000 | *Peltoeurydesmus biconicus* (1.000); *Leptodesmus marginatus* (1.000) |
|  | *c 1* | 1 | 2 | 7 | 5.666667 | *Eurydesmus baguassuensis* (1.000); *Dialysogon jucundus* (1.000); *Leptodesmus dentellus* (0.778); *Leptodesmus vagans* (0.778); *Sandalodesmus gasparae* (0.778); *Brasilodesmus paulistus paulistus* (0.556); *Henrisaussurea ramosa* (0.778) |
|  | *c 2* | 2 | 4 | 5 | 3.761719 | *Alocodesmus intermedius* (0.750); *Biporodesmus armatus* (0.762); *Biporodesmus planus* (0.750); *Plusioporodesmus bellicosus* (0.750); *Trichomorpha virgata* (0.750) |
|  | *c 3* | 3 | 2 | 7 | 7.000000 | *Achromoporus ebanoverde* (1.000); *Achromoporus magnus* (1.000); *Achromoporus martingarcia* (1.000); *Achromoporus montanus* (1.000); *Achromoporus platyurus* (1.000); *Achromoporus surieli* (1.000); *Hypselodesmus subtilissimus* (1.000) |
|  | *c 4* | 4 | 3 | 6 | 5.000000 | *Chondrodesmus atrophus* (0.833); *Chondrodesmus singularis* (0.833); *Trichomorpha capillata* (0.833); *Trichomorpha crucicola* (0.833); *Trichomorpha esulcata* (0.833); *Trichomorpha panamica* (0.833) |
|  | *c 5* | 5 | 6 | 7 | 4.750000 | *Obiricodesmus rupestris* (0.667); *Atlantodesmus teresa* (0.750); *Hoffmanopeltis contiger* (0.667); *Odontopeltis anchisteus* (0.667); *Odontopeltis conspersus* (0.667); *Odontopeltis tiradentes* (0.667); *Rupidesmus ruber* (0.667) |
|  | *c 6* | 6 | 5 | 23 | 17.400000 | *Colombodesmus catharus* (0.800); *Colombodesmus lygrus* (0.800); *Cormodesmus hirrutellus* (0.800); *Chondrodesmus attemsi* (0.480); *Chondrodesmus plataleus plataleus* (0.420); *Chondrodesmus rugosior* (0.800); *Chondrodesmus tamocalanus* (0.900); *Chondrodesmus virgatus frater* (0.800); *Leptodesmus flagellatus* (0.700); *Leptodesmus propinquus* (0.700); *Alassodesmus reductus* (0.800); *Trachelodesmus ancylophor* (0.800); *Trachelodesmus angulatus* (0.800); *Trichomorpha angulella* (0.800); *Trichomorpha eusema* (0.850); *Trichomorpha eutyla* (0.850); *Trichomorpha hoffmani* (0.700); *Trichomorpha lamottei* (0.700); *Trichomorpha paurothrix* (0.800); *Trichomorpha rugosella* (0.800); *Trichomorpha setosior* (0.800); *Trichomorpha spinosa* (0.700); *Trichomorpha tuberculosa* (0.800) |
|  | *c 7* | 7 | 2 | 2 | 2.000000 | *Eucordyloporus cornuatus* (1.000); *Scaptodesmus dentatus* (1.000) |
|  | *c 8* | 8 | 2 | 4 | 3.600000 | *Leptodesmus pubescens* (0.800); *Brasilodesmus paulistoides* (0.800); *Brasilodesmus paulistus meridionalis* (1.000); *Strongylomorpha araucariae* (1.000) |
|  | *c 9* | 9 | 5 | 17 | 12.569231 | *Eurydesmus alcatrazensis* (0.700); *Eurydesmus baguassuensis* (0.700); *Dialysogon jucundus* (0.700); *Leptodesmus dentellus* (0.800); *Leptodesmus forceps* (0.700); *Leptodesmus pubescens* (0.800); *Leptodesmus vagans* (0.800); *Sandalodesmus gasparae* (0.800); *Brasilodesmus catharinensis* (0.592); *Brasilodesmus decipiens* (0.700); *Brasilodesmus paulistoides* (0.800); *Brasilodesmus paulistus meridionalis* (0.700); *Brasilodesmus paulistus paulistus* (0.900); *Strongylomorpha araucariae* (0.700); *Strongylomorpha volutatus* (0.700); *Atlantodesmus itapurensis* (0.677); *Henrisaussurea ramosa* (0.800) |
|  | *c 10* | 10 | 3 | 5 | 3.796296 | *Arthrosolaenomeris pantanalensis* (0.463); *Arthrosolaenomeris caipora* (0.833); *Arthrosolaenomeris iara* (0.833); *Telonychopus klossae* (0.833); *Dioplosternus salvatrix* (0.833) |
|  | *c 11* | 11 | 4 | 3 | 2.375000 | *Chondrodesmus acuticollis* (0.875); *Chondrodesmus allenae* (0.750); *Chondrodesmus montanus* (0.750) |
|  | *c 12* | 12 | 4 | 3 | 2.250000 | *Camptomorpha orites* (0.750); *Inconus brunnior* (0.750); *Inconus lissus* (0.750) |
|  | *c 13* | 13 | 5 | 3 | 2.250000 | *Anisodesmus cerasinus* (0.700); *Cheirodesmus discolor* (0.700); *Tylodesmus liberiensis calathus* (0.850) |
|  | *c 14* | 14, 28 | 8 | 3 | 2.300000 - 2.500000 | *Basacantha tuberculifer* (0.700-0.833); *Mesodesmus robustus* (0.700-0.833); *Neocordyloporus falcatus* (0.833-0.900) |
|  | *c 15* | 15 | 4 | 3 | 2.187500 | *Zigwadesmus guiananus* (0.688); *Priodesmus acus acus* (0.750); *Priodesmus papillosus* (0.750) |
|  | *c 16* | 16 | 4 | 5 | 3.750000 | *Biporodesmus dentatus* (0.750); *Alyssa kalobata* (0.750); *Leptodesmus levis* (0.750); *Camptomorpha dorsalis* (0.750); *Guayapeltis witti* (0.750) |
|  | *c 17* | 17 | 4 | 8 | 6.000000 | *Eurydesmus aguirrei* (0.750); *Peltoeurydesmus biconicus* (0.750); *Obiricodesmus rupestris* (0.750); *Leptodesmus marginatus* (0.750); *Hoffmanopeltis contiger* (0.750); *Odontopeltis conspersus* (0.750); *Odontopeltis tiradentes* (0.750); *Rupidesmus ruber* (0.750) |
|  | *c 18* | 18 | 4 | 5 | 3.275000 | *Amphelictogon hoffmani* (0.875); *Amphelictogon loomisi* (0.750); *Amphelictogon strumosus* (0.750); *Amphelictogon subterraneus subterraneus* (0.450); *Amphelictogon subterraneus bahamiensis* (0.450) |
|  | *c 19* | 19 | 4 | 6 | 4.312500 | *Eucordyloporus cornuatus* (0.750); *Mesodesmus roccatii* (0.750); *Mesodesmus rugifer* (0.563); *Morphotelus mareesi* (0.750); *Morphotelus rosselati* (0.750); *Scaptodesmus dentatus* (0.750) |
|  | *c 20* | 20 | 2 | 3 | 2.500000 | *Alocodesmus gracilicornis* (1.000); *Chondrodesmus plataleus plataleus* (0.500); *Trachelodesmus uncinatus* (1.000) |
|  | *c 21* | 21 | 4 | 15 | 11.318182 | *Eurydesmus alcatrazensis* (0.750); *Eurydesmus baguassuensis* (0.750); *Dialysogon jucundus* (0.750); *Leptodesmus dentellus* (0.875); *Leptodesmus forceps* (0.750); *Leptodesmus pubescens* (0.614); *Leptodesmus vagans* (0.875); *Sandalodesmus gasparae* (0.875); *Brasilodesmus catharinensis* (0.614); *Brasilodesmus decipiens* (0.750); *Brasilodesmus paulistoides* (0.614); *Brasilodesmus paulistus paulistus* (1.000); *Strongylomorpha volutatus* (0.750); *Atlantodesmus itapurensis* (0.477); *Henrisaussurea ramosa* (0.875) |
|  | *c 22* | 22 | 3 | 4 | 3.000000 | *Cryptoporatia verrucosa* (0.833); *Diaphorodesmus dorsicornis* (0.333); *Paracordyloporus (Paracordyloporus) porati* (1.000); *Scaptodesmus granulosa* (0.833) |
|  | *c 23* | 23 | 3 | 4 | 3.196970 | *Arthrosolaenomeris chapadensis* (0.530); *Euthydesmus acicarina* (0.833); *Leiodesmus orlandi* (0.833); *Leiodesmus validus* (1.000) |
|  | *c 24* | 24 | 2 | 2 | 2.000000 | *Sandalodesmus salvadorii* (1.000); *Strongylomorpha bohlsi* (1.000) |
|  | *c 25* | 25 | 2 | 3 | 2.777778 | *Euthydesmus acicarina* (1.000); *Leiodesmus orlandi* (1.000); *Leiodesmus validus* (0.778) |
|  | *c 26* | 26 | 5 | 5 | 3.900000 | *Cryptoporatia verrucosa* (0.700); *Diaphorodesmus dorsicornis* (1.000); *Paracordyloporus (Paracordyloporus) dilatatus* (0.700); *Paracordyloporus (Paracordyloporus) porati* (0.800); *Scaptodesmus granulosa* (0.700) |
|  | *c 27* | 27 | 3 | 3 | 2.666667 | *Lithobiodesmus xenoporus* (0.833); *Rhicnostethus rondoni* (0.833); *Rondonaria schubarti* (1.000) |
|  | *c 28* | 29 | 2 | 3 | 3.000000 | *Biporodesmus dentatus* (1.000); *Leptodesmus levis* (1.000); *Guayapeltis witti* (1.000) |
|  | *c 29* | 30 | 2 | 3 | 3.000000 | *Chondrodesmus singularis* (1.000); *Trichomorpha crucicola* (1.000); *Trichomorpha esulcata* (1.000) |
|  | *c 30* | 31 | 2 | 8 | 7.500000 | *Amphelictogon alayoni* (1.000); *Amphelictogon couloni* (1.000); *Amphelictogon cubanus* (0.500); *Amphelictogon obscurus* (1.000); *Amphelictogon propinquus* (1.000); *Amphelictogon thomasi* (1.000); *Amphelictogon turquinensis* (1.000); *Plicatodesmus turquino* (1.000) |
|  | *c 31* | 32 | 2 | 3 | 3.000000 | *Arthrosolaenomeris caipora* (1.000); *Arthrosolaenomeris iara* (1.000); *Dioplosternus salvatrix* (1.000) |
|  | *c 32* | 33 | 4 | 12 | 9.187500 | Caraibodesmus criniger (55): (0.750)  58 Caraibodesmus mammatus (58): (0.750)  428 Amphelictogon alayoni (428): (0.750)  431 Amphelictogon couloni (431): (0.750)  432 Amphelictogon cubanus (432): (0.938)  443 Amphelictogon obscurus (443): (0.750)  445 Amphelictogon propinquus (445): (0.750)  453 Amphelictogon thomasi (453): (0.750)  454 Amphelictogon turquinensis (454): (0.750)  484 Cubodesmus prominens (484): (0.750)  504 Granmadesmus minor (504): (0.750)  561 Plicatodesmus turquino (561): (0.750) |
|  | *c 33* | 34 | 3 | 11 | 9.466667 | *Eurydesmus alcatrazensis* (0.833); *Eurydesmus baguassuensis* (0.833); *Dialysogon jucundus* (0.833); *Leptodesmus dentellus* (1.000); *Leptodesmus forceps* (0.833); *Leptodesmus vagans* (1.000); *Sandalodesmus gasparae* (1.000); *Brasilodesmus paulistus paulistus* (0.800); *Strongylomorpha volutatus* (0.833); *Atlantodesmus itapurensis* (0.500); *Henrisaussurea ramosa* (1.000) |
|  | *c 34* | 35 | 2 | 4 | 4.000000 | *Obiricodesmus rupestris* (1.000); *Hoffmanopeltis contiger* (1.000); *Odontopeltis conspersus* (1.000); *Rupidesmus ruber* (1.000) |
|  | *c 35* | 36 | 2 | 3 | 3.000000 | *Chondrodesmus atrophus* (1.000); *Trichomorpha capillata* (1.000); *Trichomorpha panamica* (1.000) |
|  | *c 36* | 37 | 2 | 8 | 6.500000 | *Eurydesmus alcatrazensis* (1.000); *Leptodesmus dentellus* (0.750); *Leptodesmus forceps* (1.000); *Leptodesmus vagans* (0.750); *Sandalodesmus gasparae* (0.750); *Brasilodesmus paulistus paulistus* (0.500); *Strongylomorpha volutatus* (1.000); *Henrisaussurea ramosa* (0.750) |
|  | *c 37* | 38 | 2 | 4 | 4.000000 | *Alocodesmus intermedius* (1.000); *Biporodesmus planus* (1.000); *Plusioporodesmus bellicosus* (1.000); *Trichomorpha virgata* (1.000) |
|  | *c 38* | 39 | 2 | 2 | 2.000000 | *Anisodesmus cerasinus* (1.000); *Cheirodesmus discolor* (1.000) |
|  | *c 39* | 40 | 4 | 17 | 15.000000 | *Colombodesmus catharus* (0.875); *Colombodesmus lygrus* (0.875); *Cormodesmus hirrutellus* (0.875); *Chondrodesmus nobilis* (0.750); *Chondrodesmus rugosior* (0.875); *Chondrodesmus tamocalanus* (1.000); *Chondrodesmus virgatus frater* (0.875); *Alassodesmus reductus* (0.875); *Trachelodesmus ancylophor* (0.875); *Trachelodesmus angulatus* (0.875); *Trichomorpha angulella* (0.875); *Trichomorpha eusema* (0.938); *Trichomorpha eutyla* (0.938); *Trichomorpha paurothrix* (0.875); *Trichomorpha rugosella* (0.875); *Trichomorpha setosior* (0.875); *Trichomorpha tuberculosa* (0.875) |
| 4° x 4° | *c 0* | 0 | 4 | 4 | 3.125000 | *Leptherpum capiberibei* (0.750); *Priodesmus acus acus* (0.750); *Priodesmus papillosus* (0.750); *Stenonia coralloides* (0.875) |
|  | *c 1* | 1 | 4 | 3 | 2.062500 | *Amphelictogon hoffmani* (0.750); *Amphelictogon subterraneus subterraneus* (0.563); *Amphelictogon subterraneus pinetorum* (0.750) |
|  | *c 2* | 2 | 2 | 3 | 2.333333 | *Diaphorodesmus dorsicornis* (0.667); *Paracordyloporus (Paracordyloporus) porati* (0.667); *Scaptodesmus granulosa* (1.000) |
|  | *c 3* | 3 | 4 | 4 | 2.785714 | *Amphelictogon hoffmani* (0.750); *Amphelictogon subterraneus subterraneus* (0.536); *Amphelictogon subterraneus dolius* (0.750); *Amphelictogon subterraneus pinetorum* (0.750) |
|  | *c 4* | 4 | 3 | 5 | 4.166667 | *Eucordyloporus cornuatus* (0.833); *Mesodesmus roccatii* (0.833); *Mesodesmus rugifer* (0.833); *Pimodesmus pallidus* (0.833); *Scaptodesmus dentatus* (0.833) |
|  | *c 5* | 5 | 5 | 6 | 3.946154 | *Arthrosolaenomeris chapadensis* (0.592); *Arthrosolaenomeris saci* (0.700); *Iguazus ornithopus* (0.377); *Leiodesmus orlandi* (0.700); *Leiodesmus postillonus* (0.677); *Leiodesmus validus* (0.900) |
|  | *c 6* | 6 | 4 | 4 | 2.910714 | *Leptodesmus ringueleti* (0.875); *Platinodesmus argentineus* (0.750); *Leiodesmus major* (0.536); *Odontopeltis proxima* (0.750) |
|  | *c 7* | 7 | 3 | 9 | 7.291667 | *Chondrodesmus attemsi* (0.625); *Chondrodesmus plataleus plataleus* (0.833); *Chondrodesmus voglii* (0.833); *Leptodesmus flagellatus* (0.833); *Leptodesmus propinquus* (0.833); *Lepturodesmus meinerti* (0.833); *Trichomorpha hoffmani* (0.833); *Trichomorpha lamottei* (0.833); *Trichomorpha spinosa* (0.833) |
|  | *c 8* | 8 | 3 | 7 | 5.333333 | *Leptodesmus pubescens* (1.000); *Brasilodesmus decipiens* (0.833); *Brasilodesmus paulistoides* (0.833); *Brasilodesmus paulistus meridionalis* (0.833); *Brasilodesmus paulistus paulistus* (0.667); *Strongylomorpha araucariae* (0.833); *Atlantodesmus itapurensis* (0.333) |
|  | *c* 9 | 9 | 4 | 5 | 3.500000 | *Arthrosolaenomeris chapadensis* (0.625); *Arthrosolaenomeris saci* (0.750); *Iguazus ornithopus* (0.375); *Leiodesmus orlandi* (0.750); *Leiodesmus validus* (1.000) |
|  | *c 10* | 10 | 2 | 4 | 3.500000 | *Leptodesmus pubescens* (0.750); *Brasilodesmus decipiens* (1.000); *Brasilodesmus paulistoides* (1.000); *Brasilodesmus paulistus paulistus* (0.750) |
|  | *c 11* | 11 | 3 | 4 | 3.333333 | *Alyssa kalobata* (0.833); *Chondrodesmus armatus* (0.833); *Camptomorpha dorsalis* (0.833); *Camptomorpha perproxima* (0.833) |
|  | *c 12* | 12 | 2 | 5 | 4.055556 | *Abiliodesmus planaltensis* (1.000); *Dialysogon jucundus* (0.944); *Leptodesmus tridentatus* (1.000); *Sandalodesmus gasparae* (0.778); *Atlantodesmus itapurensis* (0.333) |
|  | *c 13* | 13 | 2 | 2 | 2.000000 | *Chondrodesmus voglii* (1.000); *Lepturodesmus meinerti* (1.000) |
|  | *c 14* | 14 | 2 | 5 | 4.750000 | *Chondrodesmus attemsi* (0.750); *Chondrodesmus nobilis* (1.000); *Chondrodesmus tamocalanus* (1.000); *Trichomorpha eusema* (1.000); *Trichomorpha eutyla* (1.000) |
|  | *c 15* | 15 | 4 | 4 | 2.700000 | *Amphelictogon hoffmani* (0.750); *Amphelictogon subterraneus subterraneus* (0.450); *Amphelictogon subterraneus bahamiensis* (0.750); *Amphelictogon subterraneus dolius* (0.750) |
|  | *c 16* | 16 | 5 | 7 | 4.390909 | *Arthrosolaenomeris pantanalensis* (0.700); *Arthrosolaenomeris caipora* (0.700); *Arthrosolaenomeris iara* (0.700); *Dioplosternus salvatrix* (0.700); *Leiodesmus orlandi* (0.700); *Leiodesmus postillonus* (0.445); *Leiodesmus validus* (0.445) |
|  | *c 17* | 17 | 5 | 5 | 3.433333 | *Leptherpum capiberibei* (0.700); *Leptherpum carinovatum* (0.533); *Priodesmus acus acus* (0.700); *Priodesmus papillosus* (0.700); *Stenonia coralloides* (0.800) |
|  | *c 18* | 18 | 3 | 3 | 2.500000 | *Lipodesmus limbata limbata* (0.833); *Morphotelus rotundalatus* (0.833); *Pimodesmus numerosus* (0.833) |
|  | *c 19* | 19, 35 | 7 | 3 | 2.333333 | *Basacantha decora* (0.583-0.667); *Lipodesmus limbata rubripes* (0.875-1.000); *Paracordyloporus (Specioporus) speciosus* (0.667-0.875) |
|  | *c 20* | 20 | 3 | 22 | 16.845238 | *Alocodesmus mammatus* (0.833); *Eurydesmus alcatrazensis* (0.833); *Eurydesmus angulatus* (0.833); *Cornalatus permutatus* (0.833); *Obiricodesmus terrigena* (0.833); *Leptodesmus carneus* (0.833); *Leptodesmus dentellus* (0.833); *Leptodesmus forceps* (0.833); *Leptodesmus segadasi-viannai* (0.833); *Leptodesmus therezopolis* (0.833); *Leptodesmus ustus* (0.833); *Leptodesmus vagans* (0.833); *Sandalodesmus gasparae* (0.595); *Brasilodesmus paulistus paulistus* (0.595); *Strongylomorpha organorum* (0.833); *Strongylomorpha vanvolxemi* (0.833); *Strongylomorpha volutatus* (0.833); *Atlantodesmus itapurensis* (0.119); *Henrisaussurea corcovadis* (0.833); *Henrisaussurea ramosa* (0.833); *Heptoporodesmus alpinus* (0.833); *Odontopeltis tiradentes* (0.536) |
|  | *c 21* | 21 | 4 | 6 | 4.500000 | *Eucordyloporus cornuatus* (0.750); *Mesodesmus roccatii* (0.750); *Mesodesmus rugifer* (0.750); *Pimodesmus pallidus* (0.750); *Pimodesmus rubripes* (0.750); *Scaptodesmus dentatus* (0.750) |
|  | *c 22* | 22 | 5 | 4 | 2.800000 | *Anisodesmus cerasinus* (0.700); *Cheirodesmus discolor* (0.700); *Prepodesmus quadrilobatus* (0.700); *Tylodesmus liberiensis calathus* (0.700) |
|  | *c 23* | 23 | 5 | 17 | 12.800000 | *Achromoporus atrechoensis* (0.700); *Achromoporus ebanoverde* (0.900); *Achromoporus elegans* (0.700); *Achromoporus magnus* (0.900); *Achromoporus martingarcia* (0.700); *Achromoporus montanus* (0.900); *Achromoporus platyurus* (0.900); *Achromoporus surieli* (0.800); *Amphelictogon alayoni* (0.700); *Amphelictogon cubanus* (0.700); *Amphelictogon dentatus* (0.700); *Amphelictogon obscurus* (0.700); *Amphelictogon pallidipes* (0.700); *Cubodesmus prominens* (0.700); *Cubodesmus ramsdeni* (0.700); *Hypselodesmus assoi* (0.700); *Hypselodesmus subtilissimus* (0.700) |
|  | *c 24* | 24 | 3 | 11 | 9.333333 | *Chondrodesmus attemsi* (1.000); *Chondrodesmus nobilis* (0.833); *Chondrodesmus plataleus plataleus* (0.833); *Chondrodesmus tamocalanus* (0.833); *Leptodesmus flagellatus* (0.833); *Leptodesmus propinquus* (0.833); *Trichomorpha eusema* (0.833); *Trichomorpha eutyla* (0.833); *Trichomorpha hoffmani* (0.833); *Trichomorpha lamottei* (0.833); *Trichomorpha spinosa* (0.833) |
|  | *c 25* | 25 | 3 | 4 | 3.333333 | *Alocodesmus angustatus* (0.833); *Chondrodesmus singularis* (0.833); *Trichomorpha crucicola* (0.833); *Trichomorpha esulcata* (0.833) |
|  | *c 26* | 26 | 3 | 3 | 2.500000 | *Sandalodesmus bertonii* (0.833); *Sandalodesmus salvadorii* (0.833); *Strongylomorpha bohlsi* (0.833) |
|  | *c 27* | 27 | 3 | 3 | 2.190476 | *Leiodesmus major* (1.000); *Leiodesmus postillonus* (0.357); *Odontopeltis proxima* (0.833) |
|  | *c 28* | 28 | 2 | 2 | 2.000000 | *Morphotelus rotundalatus* (1.000); *Pimodesmus numerosus* (1.000) |
|  | *c 29* | 29 | 2 | 3 | 3.000000 | *Arthrosolaenomeris caipora* (1.000); *Arthrosolaenomeris iara* (1.000); *Dioplosternus salvatrix* (1.000) |
|  | *c 30* | 30 | 2 | 3 | 3.000000 | *Lithobiodesmus xenoporus* (1.000); *Rhicnostethus rondoni* (1.000); *Rondonaria schubarti* (1.000) |
|  | *c 31* | 31 | 3 | 7 | 6.166667 | *Cordyloconus vitiosus* (0.833); *Cryptoporatia verrucosa* (0.833); *Diaphorodesmus dorsicornis* (1.000); *Neocordyloporus longipes* (0.833); *Paracordyloporus (Paracordyloporus) porati* (1.000); *Scaptodesmus granulosa* (0.833); *Scaptodesmus porati (0.833)* |
|  | *c 32* | 32 | 2 | 2 | 2.000000 | *Priodesmus acus acus* (1.000); *Priodesmus papillosus* (1.000) |
|  | *c 33* | 33 | 2 | 8 | 4.400000 | *Achromoporus ebanoverde* (0.200); *Achromoporus heteromus* (1.000); *Achromoporus magnus* (0.200); *Achromoporus montanus* (0.200); *Achromoporus platyurus* (0.200); *Achromoporus surieli* (0.600); *Ebanodesmus victori* (1.000); *Hypselodesmus bicolor* (1.000) |
|  | *c 34* | 34 | 2 | 2 | 2.000000 | *Afolabina togoensis* (1.000); *Anisodesmus tubulatus* (1.000) |
|  | *c 35* | 36 | 2 | 6 | 5.333333 | *Cordyloconus vitiosus* (1.000); *Cryptoporatia verrucosa* (1.000); *Diaphorodesmus dorsicornis* (0.667); *Neocordyloporus longipes* (1.000); *Paracordyloporus (Paracordyloporus) porati* (0.667); *Scaptodesmus porati* (1.000) |
|  | *c 36* | 37 | 2 | 5 | 5.000000 | *Alocodesmus intermedius* (1.000); *Biporodesmus planus* (1.000); *Plusioporodesmus bellicosus* (1.000); *Chondrodesmus convexus* (1.000); *Trichomorpha virgata* (1.000) |
|  | *c 37* | 38 | 3 | 7 | 5.333333 | *Abiliodesmus planaltensis* (0.833); *Dialysogon jucundus* (0.795); *Leptodesmus geniculatus* (0.917); *Leptodesmus tridentatus* (0.833); *Sandalodesmus gasparae* (0.682); *Atlantodesmus itapurensis* (0.636); *Iguazus ornithopus* (0.636) |
|  | *c 38* | 39 | 2 | 4 | 4.000000 | *Eucordyloporus cornuatus* (1.000); *Mesodesmus roccatii* (1.000); *Mesodesmus rugifer* (1.000); *Scaptodesmus dentatus* (1.000) |
|  | *c 39* | 40 | 4 | 13 | 10.875000 | *Achromoporus atrechoensis* (0.750); *Achromoporus ebanoverde* (1.000); *Achromoporus elegans* (0.750); *Achromoporus heteromus* (0.750); *Achromoporus magnus* (1.000); *Achromoporus martingarcia* (0.750); *Achromoporus montanus* (1.000); *Achromoporus platyurus* (1.000); *Achromoporus surieli* (0.875); *Ebanodesmus victori* (0.750); *Hypselodesmus assoi* (0.750); *Hypselodesmus bicolor* (0.750); *Hypselodesmus subtilissimus* (0.750) |
|  | *c 40* | 41 | 2 | 3 | 3.000000 | *Chondrodesmus armatus* (1.000); *Camptomorpha dorsalis* (1.000); *Camptomorpha perproxima* (1.000) |
|  | *c 41* | 42 | 2 | 7 | 6.666667 | *Chondrodesmus attemsi* (0.667); *Chondrodesmus plataleus plataleus* (1.000); *Leptodesmus flagellatus* (1.000); *Leptodesmus propinquus* (1.000); *Trichomorpha hoffmani* (1.000); *Trichomorpha lamottei* (1.000); *Trichomorpha spinosa* (1.000) |
|  | *c 42* | 43 | 2 | 2 | 2.000000 | *Trichomorpha folia* (1.000); *Trichomorpha hyla* (1.000) |
|  | *c 43* | 44 | 2 | 7 | 4.714286 | *Achromoporus atrechoensis* (1.000); *Achromoporus ebanoverde* (0.429); *Achromoporus elegans* (1.000); *Achromoporus magnus* (0.429); *Achromoporus montanus* (0.429); *Achromoporus platyurus* (0.429); *Hypselodesmus assoi* (1.000) |
|  | *c 44* | 45 | 2 | 3 | 3.000000 | *Chondrodesmus singularis* (1.000); *Trichomorpha crucicola* (1.000); *Trichomorpha esulcata* (1.000) |
|  | *c 45* | 46 | 2 | 13 | 12.500000 | *Alocodesmus mammatus* (1.000); *Eurydesmus angulatus* (1.000); *Cornalatus permutatus* (1.000); *Obiricodesmus terrigena* (1.000); *Leptodesmus carneus* (1.000); *Leptodesmus segadasi-viannai* (1.000); *Leptodesmus therezopolis* (1.000); *Leptodesmus ustus* (1.000); *Strongylomorpha organorum* (1.000); *Strongylomorpha vanvolxemi* (1.000); *Henrisaussurea corcovadis* (1.000); *Heptoporodesmus alpinus* (1.000); *Odontopeltis tiradentes* (0.500) |
|  | *c 46* | 47 | 2 | 2 | 2.000000 | *Sandalodesmus salvadorii* (1.000); *Strongylomorpha bohlsi* (1.000) |
